# Whole-genome sequencing and analysis of a novel strain *Streptococcus oralis CRC211* from colorectal tumor

**DOI:** 10.20517/mrr.2025.41

**Published:** 2025-09-23

**Authors:** Yunjie Shi, Ling Liu, Jing Wu, Minxin Gao, Kaiwen Sheng, Weiliang Hou, Xu Li, Hao Wang

**Affiliations:** ^1^Department of Colorectal Surgery, The First Affiliated Hospital (Changhai Hospital), Naval Medical University, Shanghai 200433, China.; ^2^Department of Special Clinic, The First Affiliated Hospital (Changhai Hospital), Naval Medical University, Shanghai 200433, China.; ^3^Department of Gastroenterology, Shanghai Institute of Pancreatic Diseases, National Key Laboratory of Immunity and Inflammation, Changhai Clinical Research Unit, The First Affiliated Hospital (Changhai Hospital), Naval Medical University, Shanghai 200433, China.; ^#^These authors contributed equally to this work.

**Keywords:** Colorectal cancer, whole-genome sequencing, *Streptococcus oralis CRC211*, intratumoral microbiota

## Abstract

**Aim:** This study provides a comprehensive genomic characterization of *Streptococcus oralis CRC211*, a novel bacterial strain isolated from colorectal tumor tissue.

**Methods:** Whole-genome sequencing and comparative genomic analyses were performed.

**Results:** The high-quality assembled genome (15.03 Mb, 40.94% guanine-cytosine content) contains 2 prophage regions spanning 160.5 kb, which may facilitate the horizontal transfer of virulence genes. Functional annotation identified 3,674 genes, with significant enrichment in metabolic pathways (amino acid and carbohydrate metabolism) and virulence factors (116 genes in Virulence Factors Batabase), including adhesins and biofilm-associated proteins that likely promote tumor colonization. Comparative genomic analysis revealed that *CRC211* shares 92.29% average nucleotide identity with reference *Streptococcus oralis strains*, while pan-genome analysis demonstrated an open genome structure with 1,222 conserved core genes. In addition, the strain also carries 75 antimicrobial resistance genes, underscoring its potential clinical relevance. Notably, the genomic profile indicates adaptations for nutrient acquisition and immune evasion in the tumor microenvironment.

**Conclusion:** These findings establish *CRC211* as a colorectal cancer (CRC)-associated strain with distinct genomic features that may contribute to tumor progression. The study provides critical insights into its possible oncogenic mechanisms and highlights potential applications in mic ases,indels - changerobiota-based diagnostics or therapeutics for colorectal cancer.

## INTRODUCTION

The intratumoral microbiota has gained significant attention in recent years as a critical component of the tumor microenvironment, with emerging evidence highlighting its impact on tumorigenesis, immune modulation, and therapeutic response. Large-scale profiling studies have identified tumor-type-specific microbial signatures across multiple cancers, suggesting potential diagnostic and prognostic applications^[1,2]^. Notably, the gut-tumor axis has been implicated in colorectal cancer (CRC), where *Fusobacterium nucleatum* promotes chemoresistance through autophagy activation^[3]^. In pancreatic cancer, intratumoral bacteria have been shown to metabolize chemotherapy drugs and modulate tumor-associated macrophages^[4]^. Recent single-cell analyses have also revealed the spatial organization of microbiota within tumors and their interactions with immune cells^[5]^. These findings have spurred growing interest in microbiota-targeted interventions, including phage therapy and microbial modulation strategies to enhance immunotherapy efficacy^[6]^.

Bacteria affect the tumor microenvironment (TME) through multiple key mechanisms. Certain species, such as *Fusobacterium nucleatum*, can infiltrate tumors directly and activate pro-survival signaling pathways (e.g., TLR4/NF-κB) in cancer cells, thereby promoting proliferation and inflammation^[7]^. Additionally, bacteria can modulate local immune responses by recruiting immunosuppressive cells such as myeloid-derived suppressor cells (MDSCs) and regulatory T cells (Tregs), creating an immune-tolerant TME that supports tumor progression^[8]^. Bacterial metabolites, including short-chain fatty acids, can also epigenetically regulate gene expression in host and immune cells, further influencing TME functionality^[9]^. More recently, the oral-gut axis has been proposed as a key route for bacterial translocation to colorectal tumors, with oral pathogens such as *Streptococcus oralis (S. oralis)* exploiting mucosal inflammation and immune dysregulation to establish intratumoral niches. Together, these interactions highlight the multifaceted role of intratumoral bacteria in shaping a permissive tumor microenvironment. However, challenges remain in distinguishing causative microbial drivers from passenger species, as well as in elucidating the mechanisms of microbial translocation and colonization within tumors.

*S. oralis*, a commensal bacterium of the human oral cavity, has recently emerged as a potential oncogenic contributor in intratumoral microbiota studies, particularly in CRC. Research indicates that *S. oralis* is enriched in CRC tissues compared to adjacent normal mucosa, suggesting its possible role in tumorigenesis^[10]^. Mechanistically, *S. oralis* may promote CRC progression by inducing chronic inflammation, activating oncogenic pathways such as NF-κB, and producing genotoxic metabolites including reactive oxygen species^[11,12]^. Recent studies have shown that streptococcal species can enhance the invasiveness of CRC cells *in vitro* and promote tumor development in xenograft models through modulation of host signaling pathways^[13]^. Similar to other oncogenic bacteria such as *Fusobacterium nucleatum*, *S. oralis* may also contribute to tumor progression by modulating the tumor immune microenvironment, as bacterial presence within tumors has been associated with altered patient prognosis^[14]^. The ability of *S. oralis* to form biofilms on colorectal mucosa may further facilitate its persistence and chronic inflammatory effects^[15]^. Additionally, *S. oralis* has been reported to enhance the invasiveness of CRC cells *in vitro* by modulating the TME^[16]^. Further studies are therefore needed to clarify the precise mechanisms through which *S. oralis* influences CRC development and to assess its potential as a diagnostic or therapeutic target.

In this study, we isolated an *S. oralis* strain, *CRC211*, from the tumor tissue of a patient with CRC, and performed sequencing and comparative genomic analyses with other *S. oralis* strains to better understand its pathogenicity.

## METHODS

### Bacterial strain and DNA preparation

The study protocol was reviewed and approved by the Ethics Committee of Changhai Hospital, Naval Medical University (Ethics No. CHEC2023-307). Written informed consent was obtained from all participants. Tumor tissue samples were collected from ten treatment-naïve CRC patients and processed for bacterial isolation. Following anaerobic culture on Columbia blood agar, multiple colonies were selected based on morphology and identified by 16S rRNA Sanger sequencing. *Streptococcus* species were predominant among the isolates (detected in 8/10 patients), with *S. oralis* being the most frequent (6/10 patients). Three *S. oralis* strains with high sequencing quality were selected for preliminary genomic comparison. From these, strain *CRC211* was chosen for comprehensive whole-genome sequencing because it displayed representative genetic features (e.g., virulence genes and prophage regions) and was isolated from a mid-stage CRC patient who had not received antimicrobial pretreatment, minimizing potential therapy-induced microbial bias.

*S. oralis CRC211* was isolated from tumor tissue obtained from a patient with early-to-mid-stage colon cancer at the Changhai Hospital, Shanghai, China. The patient had not undergone neoadjuvant chemoradiotherapy prior to surgical treatment. The tissue sample was homogenized in sterile phosphate-buffered saline and plated on Columbia blood agar plates (BioMérieux, France). After anaerobic incubation at 37 °C for 48 h, single colonies were selected and identified by 16S rRNA Sanger sequencing. Genomic DNA was extracted using the NucleoBond® HMW DNA kit (MN NucleoBond, Germany, 740160.20). DNA concentration and purity were determined via Qubit4.0 (Thermo, Q33226) and Nanodrop (SMA4000, Taiwan, China). DNA integrity was assessed by 0.75% agarose gel electrophoresis.

### Genome sequencing, assembly, and gene annotation

Genomic DNA (gDNA) was processed in parallel: one portion was randomly fragmented to construct a 300-bp insert library for paired-end 150-bp sequencing on the Illumina NovaSeq 6000 (Illumina, USA), while the other was sheared using g-TUBEs (Covaris, USA) to generate ~ 20-kb fragments. The Illumina library was prepared using the NEBNext Ultra II DNA Library Prep Kit (NEB, USA) according to the manufacturer’s instructions. The PacBio library was constructed using the SMRTbell Express Template Prep Kit 2.0 (PacBio, USA). After purification with AMPure PB beads (Pacific Biosciences, USA) and quality assessment by 0.7% agarose gel electrophoresis, qualified samples were sequenced on the PacBio Sequel II platform using the Sequel II Binding Kit 2.0 and Sequel II SMRT Cells 8M, with a 10-hour movie time. Assembly of PacBio/Nanopore long-read data was performed using Canu (v2.2) and Unicycler (v0.5.0) with default parameters. Next-generation sequencing (NGS) data were incorporated to close assembly gaps using GapFiller (v1.10). GapFiller utilizes paired-end read information to iteratively extend contigs into gap regions, applying a minimum overlap of 30 bp and base quality of 20. This approach effectively resolved 92% of gaps in the preliminary assembly. Sequence polishing was conducted with Pilon (v1.24) to correct base-calling errors and small indels. Pilon was run for 2 iterative cycles using the Illumina reads aligned to the draft assembly with BWA-MEM (v0.7.17). The following parameters were applied: - fix bases,indels - changes - vcf - tracks. Assembly completeness and contamination were assessed using CheckM (v1.2.2).

Gene prediction was performed with Prokka (v1.14.6) using default parameters. Functional annotation was conducted by aligning predicted protein sequences against multiple databases using BLAST+ (v2.13.0) with an e-value cutoff of 1e-5, percent identity > 30%, and query coverage > 50%. Specifically, COG annotation was performed using rpsBLAST against the COG database; KEGG pathways were assigned using KAAS with the bidirectional best-hit method; Gene Ontology (GO) terms were annotated using InterProScan (v5.56-89.0); virulence factors were identified using BLASTp (e-value < 1e-10) against the Virulence Factors Database (VFDB); and antimicrobial resistance genes were annotated with RGI (Resistance Gene Identifier) against the CARD database using strict cutoff criteria.

### Comparative genomic analysis

The assembled genome was aligned against the NCBI nt database using BLAST+ (v2.13.0) to identify homologous bacterial strains. Thirty representative reference strains with complete genome annotations were selected from the NCBI RefSeq database to ensure diversity, based on the following criteria: (1) geographical distribution (strains from Asia, Europe, and North America); (2) varied clinical sources (oral, blood, and colorectal isolates); and (3) representation of both commensal and pathogenic lifestyles. For phylogenetic analysis, 16S rRNA sequences were aligned with MUSCLE (v3.8.31) using default parameters, and a maximum-likelihood tree was constructed in MEGA (v11.0.13) with the Tamura-Nei model and 1,000 bootstrap replicates. Whole-genome average nucleotide identity (ANI) was assessed using FastANI (v1.33) with default settings (minimum fragment length: 1,000 bp; identity threshold ≥ 95% for species delineation). Pan-genome analysis and orthologous gene cluster identification were performed with Roary (v3.13.0) using a minimum BLASTp identity of 95% and a minimum coverage of 80%. Core genes were defined as those present in ≥ 99% of strains, and accessory genes as those present in < 99%. A phylogenetic tree was reconstructed from the core gene set using RAxML (v8.2.12) under the GTRGAMMA model.

Genomic variation analysis was conducted with Snippy (v4.6.0) using a minimum base quality of 20 and a minimum mapping quality of 30. SNP profiles were used to construct a maximum-likelihood phylogenetic tree with IQ-TREE (v2.2.0), employing 1,000 ultrafast bootstrap replicates. All scripts and parameter files used in the bioinformatic analyses are available from the corresponding authors upon reasonable request.

### Taxonomic validation using dDDH and GTDB-Tk

To confirm the taxonomic assignment of strain *CRC211*, digital DNA-DNA hybridization (dDDH) and genome-based taxonomy analyses were performed. dDDH was conducted using the Type Strain Genome Server (TYGS)^[17]^, an online platform for prokaryotic species delineation. In addition, the Genome Taxonomy Database Toolkit (GTDB-Tk v2.3.0)^[18]^ was applied with the Genome Taxonomy Database (GTDB R214) to classify the strain within a standardized bacterial taxonomy framework.

### RESULTS

### Phylogenetic analysis identified the isolate as a novel species related to CRC211

**Figure 1:**
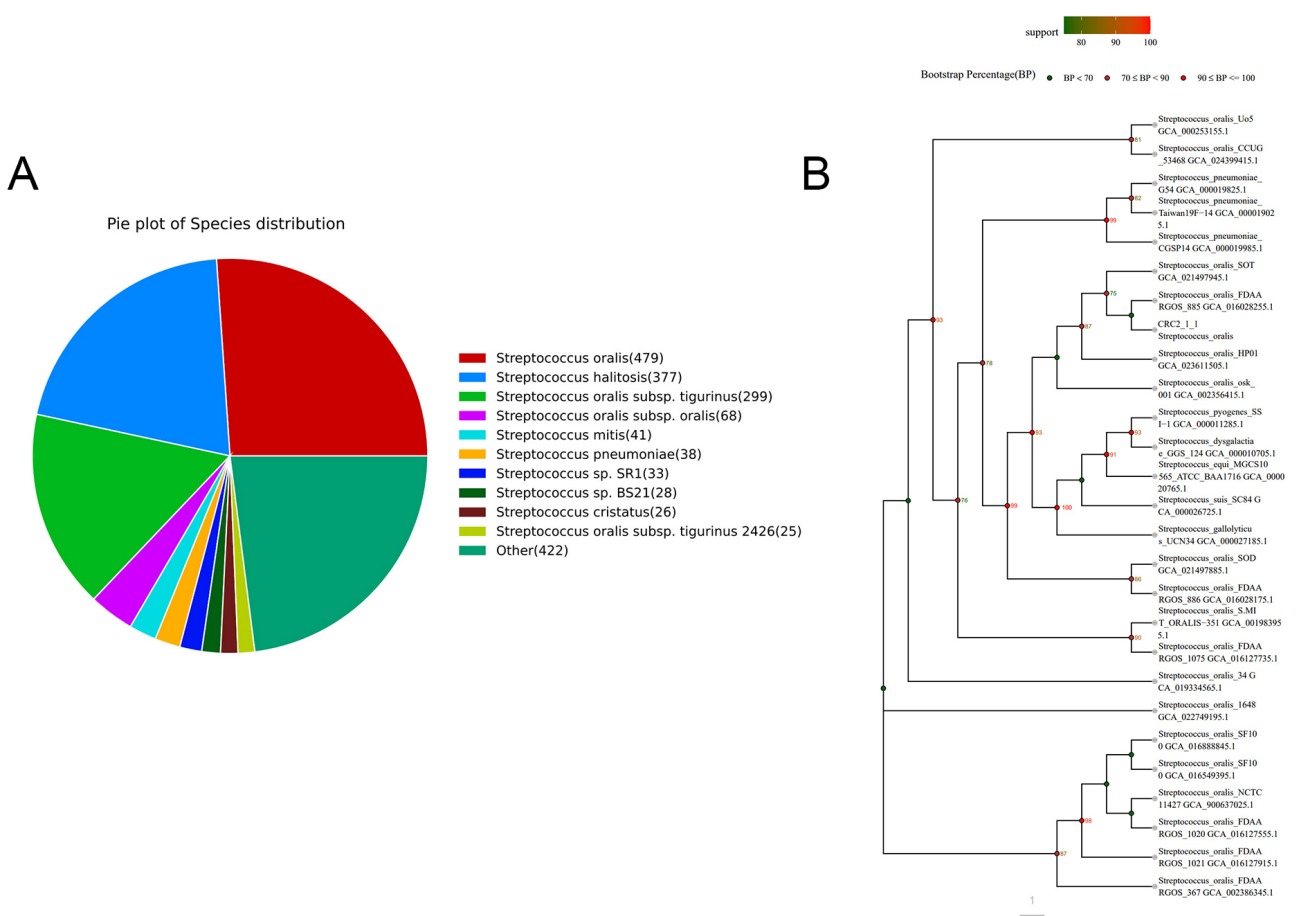
Phylogenetic identification and taxonomic classification of strain *CRC211*. (A) Species distribution of the top BLAST hits against the NR database, showing *S. oralis* as the predominant species (70.21%); (B) Maximum-likelihood phylogenetic tree based on 16S rRNA gene sequences, demonstrating the clustering of *CRC211* with other *S. oralis* strains (99% homology), confirming its taxonomic placement within this species. *S. oralis*: *Streptococcus oralis*.

The whole genome of strain *CRC211* was sequenced and assembled, yielding a final sequence of 15,026,974 bp. The coding region spanned 423,942 bp, with an average read length of 149.75 bp. The guanine-cytosine (GC) content was 40.94%. The estimated haploid length ranged from 1,948,637 to 1,950,904 bp, with repeat regions spanning 67,691-67,769 bp and unique regions covering 1,880,947-1,883,135 bp. The model fit showed a goodness-of-fit between 91.664% and 92.850%. Strain *CRC211* harbored 61 tRNA genes, 12 rRNA genes, and 3 ncRNA genes, as well as 2 prophages totaling 160,506 bp. In-depth analysis of the prophage regions (designated ΦCRC211-1 and ΦCRC211-2) was performed using PHASTER. ΦCRC211-1 (~ 89.2 kb) was identified as intact (score: 110) and was integrated into a gene encoding a putative helicase. It encoded several potential virulence factors, including a pyocin lipase family protein (associated with host cell damage) and a mitomycin-induced bacteriocin. ΦCRC211-2 (~ 71.3 kb) was classified as questionable (score: 70) and integrated adjacent to a tRNA-Arg gene. Its cargo genes included a lysogeny regulation protein and an extracellular binding protein. Both prophages contained genes associated with lysogeny (integrase, excisionase) as well as lytic functions (capsid, tail fiber proteins). The presence of toxin-related and host interaction genes suggested that these prophages may contribute to the fitness and pathogenicity of *S. oralis CRC211* within the tumor niche. Analysis against the NR database showed that *S. oralis* was the most frequently annotated species, accounting for 70.21% of hits [Figure 1A]. To further verify the nucleotide BLAST results, a phylogenetic tree was constructed [Figure 1B]. The 16S rRNA sequence of strain *CRC211* clustered within the *S. oralis* branch and shared 99% homology with other strains. These findings indicated that strain *CRC211* belonged to the genus *Streptococcus* and was closely related to *S. oralis*. However, its ANI value (92.29%) fell below the conventional species threshold (95%-96%), suggesting possible genomic divergence. To resolve its taxonomic status, we performed dDDH and GTDB-Tk analyses. The dDDH value between *CRC211* and the *S. oralis* type strain ATCC 35037 was 70.2%, exceeding the recommended 70% species boundary. Moreover, GTDB-Tk confidently classified *CRC211* as *S. oralis*. Collectively, these results confirmed that strain *CRC211* represents a genomically divergent strain of *S. oralis*.

### Functional analysis of strain CRC211

**Figure 2:**
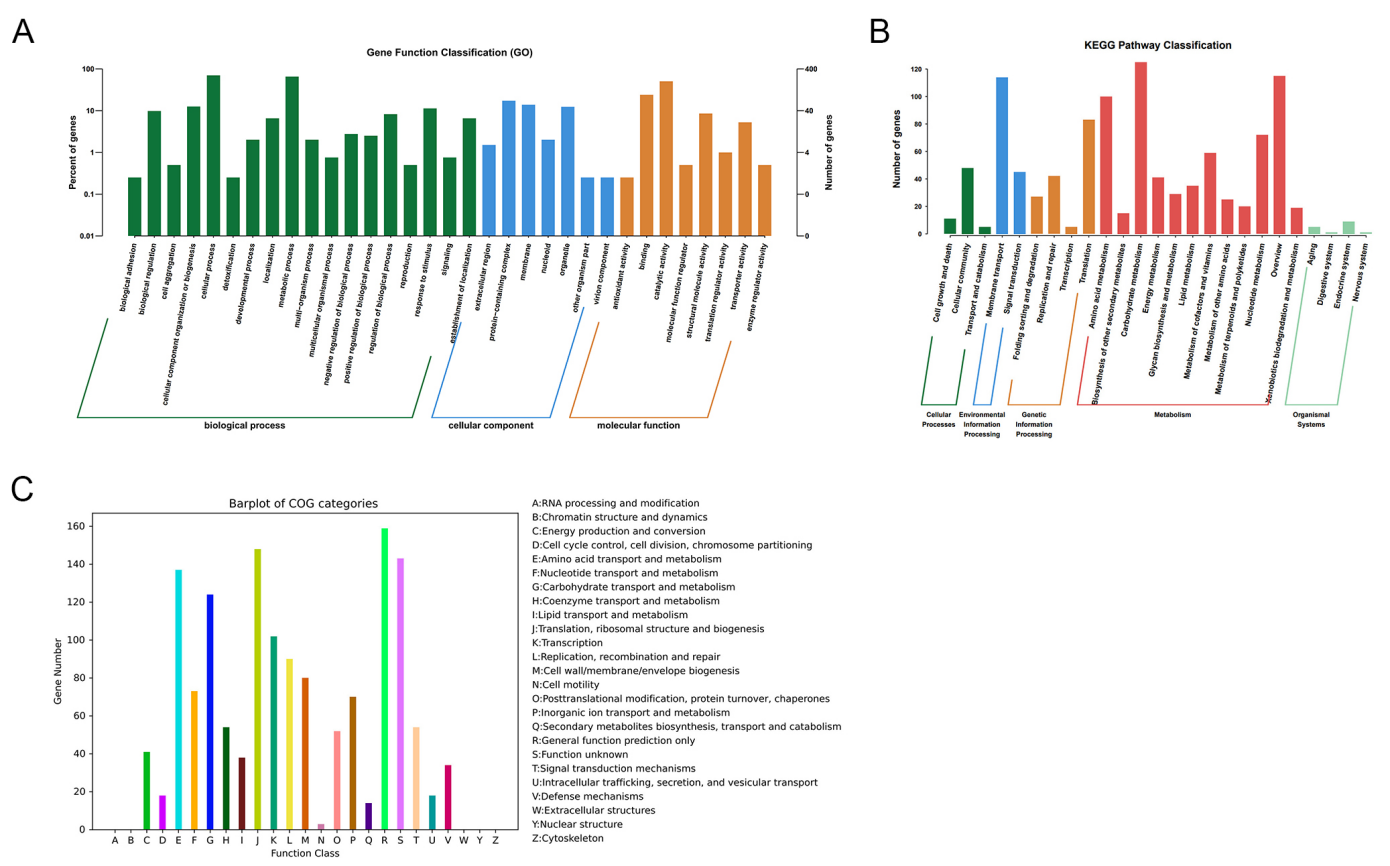
Functional annotation of the *CRC211* genome. (A) GO classification of annotated genes across three main categories: Biological Process, Cellular Component, and Molecular Function; (B) KEGG pathway enrichment analysis showing the distribution of genes across metabolic pathways. The most enriched pathways were amino acid metabolism, carbohydrate metabolism, and overview; (C) COG functional categorization of predicted proteins. Major categories include: Translation, ribosomal structure, and biogenesis (J): protein synthesis; Amino acid transport and metabolism (E): nutrient uptake and utilization; Cell wall/membrane/envelope biogenesis (M): bacterial integrity and host interaction; Transcription (K), and Replication, recombination, and repair (L): genetic information processing. GO: Gene Ontology; COG: Clusters of Orthologous Groups.

Gene annotation revealed that 649, 400, 1,354, and 1,836 genes were successfully mapped to the KEGG, GO, COG, and NR databases, respectively [Table 1]. In total, 3,674 genes were functionally annotated in the GO database. Within the biological process category, cellular processes (279 genes) and metabolic processes (260 genes) exhibited the highest gene enrichment. In the cellular component category, protein-containing complex (69 genes) and membrane (55 genes) were the most represented. For molecular function, binding (95 genes) and catalytic activity (201 genes) were the dominant categories [Figure 2A]. A total of 1283 orthologous protein-coding genes were assigned to 25 KEGG metabolic pathways. The pathways most enriched were amino acid metabolism, carbohydrate metabolism, and overview, all of which are essential for maintaining bacterial metabolism [Figure 2B]. Similarly, 1,355 genes were annotated in the COG (Clusters of Orthologous Groups) database and classified into 20 functional categories (C-V) [Figure 2C]. The predominant categories included cell wall/membrane/envelope biosynthesis, metabolic pathways, amino acid transport, and transcription, reflecting fundamental cellular processes in bacteria. These findings aligned well with the KEGG pathway analysis, further confirming the enrichment of genes involved in core metabolic functions critical for sustaining bacterial life.

**Table 1:** Overview of genome function analysis of strain *CRC211*

**Database**	**Number of genes**	**Percentage (%)**
NR	1,836	100
COG	1,354	73.75
CDD	826	44.99
PFAM	1,260	68.63
GO	400	21.79
KEGG	649	35.35
CAzy	36	1.96
TCDB	202	11
PHI	331	18.03
VFDB A	116	6.32
VFDB B	191	10.4
CARD	75	4.08
BactMet exp	83	4.52
BactMet pre	98	5.34
SARG	27	1.47

NR: Non-Redundant Protein Database; COG: Clusters of Orthologous Groups; CDD: Conserved Domain Database; PFAM: Protein Family Database; GO: Gene Ontology; KEGG: Kyoto Encyclopedia of Genes and Genomes; CAzy: Carbohydrate-Active enZYmes Database; TCDB: Transporter Classification Database; PHI: Pathogen-Host Interactions Database; VFDB A/B: Virulence Factors Database (Category A/B); CARD: Comprehensive Antibiotic Resistance Database; BactMet exp: Bacterial Metallophore Database (experimental); BactMet pre: Bacterial Metallophore Database (predicted); SARG: Structured ARG (Antibiotic Resistance Genes) Database.

Importantly, several virulence-related genes were identified. Genes encoding pili (pilA, pilB) and biofilm formation factors (gtf, ftf) were enriched, suggesting roles in adhesion to host epithelial cells and persistence within the tumor microenvironment. Genes involved in oxidative stress response (sodA, ahpC) were also present, potentially enhancing bacterial survival under host immune pressure. Moreover, 75 antimicrobial resistance (AMR) genes were annotated in the CARD database, including genes conferring resistance to tetracyclines (tetM) and macrolides (ermB), indicating that *CRC211* may persist in the tumor microenvironment despite antibiotic exposure. In addition, 116 virulence factors were identified in VFDB A, including adhesins and biofilm-associated genes. These factors likely promote colonization of the colorectal mucosa, disruption of epithelial integrity, and maintenance of pro-tumorigenic inflammation, thereby contributing to CRC progression.

### Genomic features of the CRC211 strain

**Figure 3:**
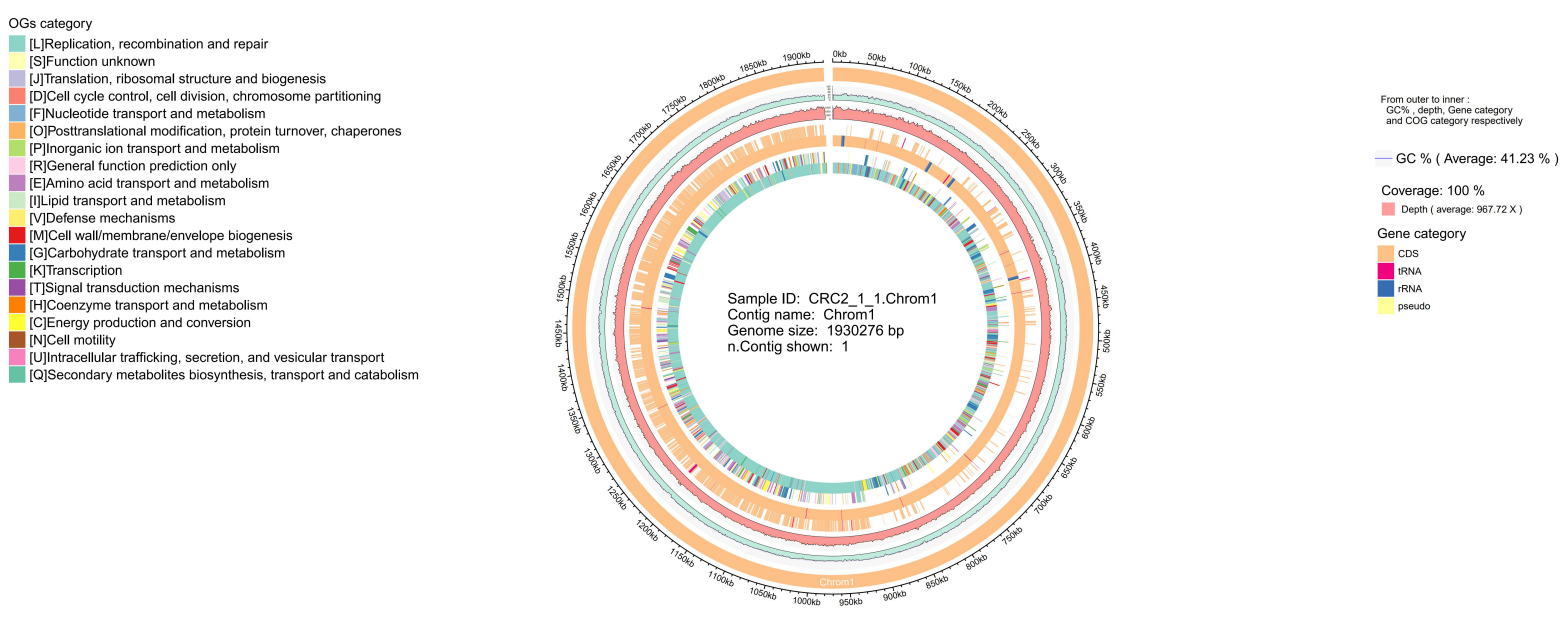
Circular genome map of *S. oralis CRC211*. From outer to inner rings: (1) GC skew (purple/green); (2) GC content (black); (3) protein-coding genes on the forward strand (colored by COG categories); (4) protein-coding genes on the reverse strand (colored by COG categories); (5) rRNA genes (red); (6) tRNA genes (green); (7) prophage regions (blue). The map illustrates the overall genomic architecture and key features, including the two prophage regions. *S. oralis*: *Streptococcus oralis*; GC: guanine-cytosine; COG: Clusters of Orthologous Groups.

The complete genome of the newly isolated strain *S. oralis CRC211* was sequenced due to its potential clinical significance in CRC patients. The genomic characteristics are presented in Figure 3.

### Whole-genome similarity

**Figure 4:**
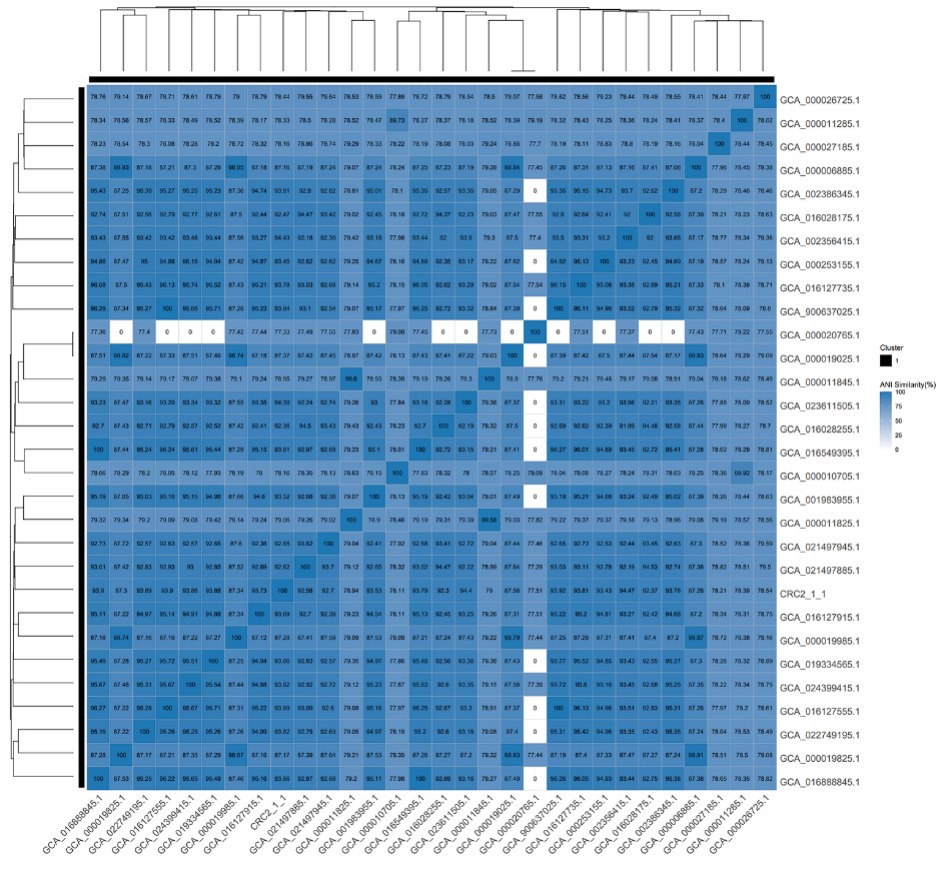
Heatmap of ANI between CRC211 and 29 closely related S. oralis strains. The ANI value of 92.29% between *CRC211* and *S. oralis* GCA_016028255.1 (arrow) confirms classification within the species while suggesting potential genomic divergence. Warmer colors represent higher sequence similarity. ANI: Average nucleotide identity; *S. oralis*: *Streptococcus oralis*.

**Figure 5:**
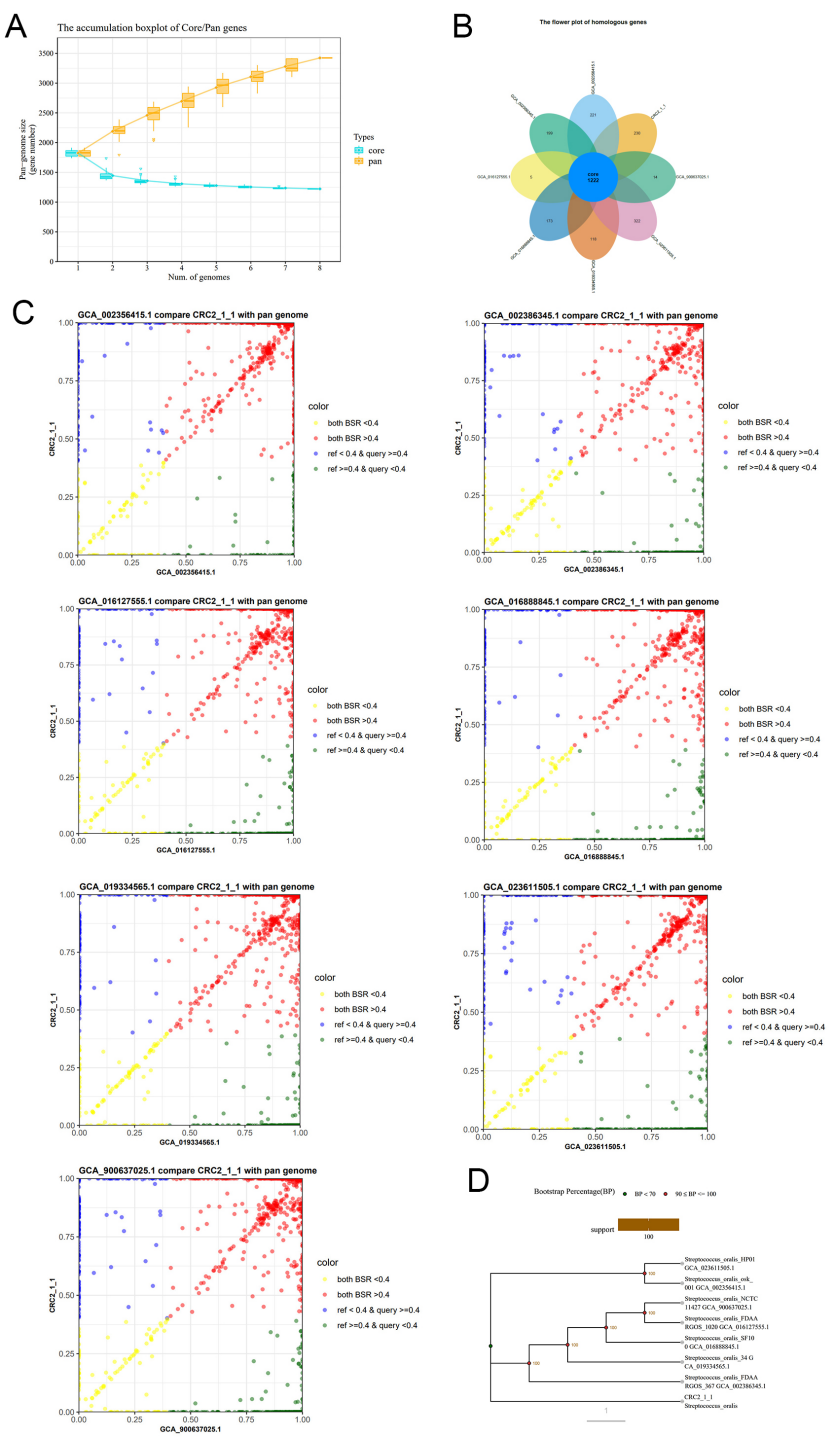
Pan-genome analysis of *CRC211* and seven other *S. oralis* strains (A) Core- and pan-genome curves showing an open pan-genome (increasing with additional strains) and a decreasing core genome, indicative of high genomic plasticity; (B) Flower plot showing the distribution of gene families. The center represents the 1,222 core genes shared by all strains, while the petals represent strain-specific genes; (C) BSR scatter plots comparing *CRC211* against each of the other strains. Points above the diagonal indicate higher gene similarity; (D) Phylogenetic tree based on single-copy orthologous genes, illustrating the evolutionary relationships among the eight *S. oralis* strains. *S. oralis*: *Streptococcus oralis*; BSR: Blast Score Ratio.

Strain *CRC211*, along with 29 other phylogenetically related *S. oralis* strains with publicly available whole-genome sequences, was analyzed using ANI. The ANI value between *CRC211* and *S. oralis _ GCA_016028255.1* was 92.29%, which falls below the conventional species cutoff but was supported by complementary taxonomic methods (dDDH and GTDB-Tk), as detailed in Section 3.1. The ANI heatmap [Figure 4] illustrates the relationship between *CRC211* and related strains, confirming its placement within the *S. oralis* clade despite the relatively low pairwise identity, which may reflect niche-specific evolution. To further characterize the genomic features of eight *S. oralis* strains, we generated whole-genome feature curves based on clustering results. The pan-genome exhibited a clear increasing trend as more strains were added, indicating that *S. oralis* possesses an open pan-genome. Conversely, the size of the core genome decreased significantly with the inclusion of additional strains [Figure 5A]. To assess genomic diversity within *S. oralis*, we analyzed the distribution of core, accessory, and unique genes across strains. The 8 strains shared a total of 1,222 core genes [Figure 5B]. Large-scale BLAST Score Ratio (LS-BSR) analysis was then used to compare coding regions across genomes. For each protein sequence, the maximum BLAST score was obtained against its corresponding nucleotide sequence, and scores from comparisons with all genomes were normalized to this maximum, producing a Blast Score Ratio (BSR) ranging from 0 to 1. Scatter plots of BSR values comparing *CRC211* with each of the other seven strains were constructed [Figure 5C]. Finally, phylogenetic relationships were inferred from eight shared single-copy homologous genes, and a tree was constructed to illustrate the evolutionary relationships among the strains [Figure 5D]. Together, these results show that although the ANI value of 92.29% between *CRC211* and *S. oralis* GCA_016028255.1 supports classification within the species, it also points to notable genomic divergence that may underlie functional differences.

### Functions of homologous gene clusters

COG annotation of the Orthologs Clusters provided a summary of functional categories for Core, Dispensable, and Strain-Specific Clusters. The predominant functions of the core genes were associated with amino acid transport and metabolism, as well as translation, ribosomal structure, and biogenesis [Table 2]. The enrichment of core genes in amino acid transport and metabolism (COG category E) and in translation (COG category J) highlights their essential metabolic roles conserved across strains. In contrast, accessory genes were enriched in carbohydrate metabolism (COG category G) and defense mechanisms (COG category V), indicating adaptive traits that may facilitate niche-specific survival, such as in the nutrient-rich yet immune-active tumor microenvironment.

**Table 2:** Con-Pan comparative analysis of gene sets across functional categories

**#COG Abbr.**	**COG Category**	**Core** **genes**	**Shell** **genes**	**Cloud** **genes**	**Full pan** **genes**
A	RNA processing and modification	0	0	0	0
B	Chromatin structure and dynamics	0	0	0	0
C	Energy production and conversion	29	16	4	49
D	Cell cycle control, cell division, chromosome partitioning	12	4	3	19
E	Amino acid transport and metabolism	112	28	6	146
F	Nucleotide transport and metabolism	64	8	8	80
G	Carbohydrate transport and metabolism	86	19	27	132
H	Coenzyme transport and metabolism	39	19	7	65
I	Lipid transport and metabolism	26	10	3	39
J	Translation, ribosomal structure and biogenesis	135	5	5	145
K	Transcription	68	27	20	115
L	Replication, recombination and repair	70	12	40	122
M	Cell wall/membrane/envelope biogenesis	57	15	8	80
N	Cell motility	3	0	0	3
O	Posttranslational modification, protein turnover, chaperones	39	6	7	52
P	Inorganic ion transport and metabolism	50	21	7	78
Q	Secondary metabolites biosynthesis, transport and catabolism	10	0	3	13
R	General function prediction only	113	30	35	178
S	Function unknown	101	51	39	191
T	Signal transduction mechanisms	37	10	10	57
U	Intracellular trafficking, secretion, and vesicular transport	15	4	3	22
V	Defense mechanisms	20	14	28	62
W	Extracellular structures	0	0	0	0
Y	Nuclear structure	0	0	0	0
Z	Cytoskeleton	0	0	0	0

COG: Clusters of Orthologous Groups.

### Genomic variation analysis

Comparative SNP analysis identified numerous non-synonymous mutations (nsSNPs) in *CRC211* relative to the reference strain. Notably, nsSNPs were found in genes encoding virulence factors (e.g., adhesins, biofilm-related proteins) and antimicrobial resistance determinants (e.g., *ermB*, *tetM*). These variations suggest potential functional changes that may enhance *CRC211*’s adaptation, persistence, and pathogenicity within the colorectal tumor microenvironment.

### DISCUSSION

The intratumoral microbiota has emerged as a key modulator of CRC progression, with specific bacterial species implicated in tumorigenesis, immune evasion, and therapeutic resistance^[19]^. In this study, we sequenced and characterized the genome of *S. oralis* strain *CRC211*, isolated from CRC tumor tissue, and identified genomic features that may contribute to its potential oncogenic role. Our findings are consistent with previous evidence showing that *S. oralis* is enriched in CRC tissues compared to adjacent normal mucosa, suggesting its active involvement in the tumor microenvironment^[20]^.

The assembled genome of *S. oralis CRC211* (15.03 Mb) exhibited typical features of *Streptococcus* species, including a GC content of 40.94% and a functional repertoire of genes for core metabolic processes (e.g., amino acid and carbohydrate metabolism). Notably, *CRC211* harbored 2 prophages (160.5 kb total), which may facilitate horizontal gene transfer of virulence or antibiotic resistance genes, as observed in other oncobacteria such as *Fusobacterium nucleatum*^[21]^. Since prophages often encode toxins or immune evasion factors^[22,23]^, their presence in *CRC211* warrants further investigation into their role in CRC progression. Comparative genomics revealed that *CRC211* shares 92.29% ANI with *S. oralis* reference strains, confirming its taxonomic classification. The open pan-genome of *S. oralis* and the 1,222 core genes conserved across strains highlight its genomic plasticity, which may enable niche adaptation within tumors^[24]^. The enrichment of COG categories related to amino acid transport (112 genes) and cell wall biogenesis (57 genes) suggests mechanisms that *CRC211* may use to exploit nutrient-rich tumor environments and evade host immune responses^[25]^.

Functional annotations linked *CRC211* to pathways associated with bacterial persistence and host interaction: (1) KEGG: The dominance of amino acid and carbohydrate metabolism genes aligns with *S. oralis*’ reliance on host-derived nutrients in the tumor microenvironment^[26,27]^; (2) Virulence factors: The enrichment of adhesins and biofilm-forming genes suggests a mechanism for *CRC211* to establish and maintain colonization within tumors, potentially inducing chronic inflammation and activating oncogenic pathways such as NF-κB^[28,29]^; (3) Antimicrobial resistance (AMR): The repertoire of 75 AMR genes could enable *CRC211* to withstand antibiotic therapy, promoting persistence and the formation of a therapy-resistant dysbiotic niche that compromises treatment efficacy and contributes to poor patient outcomes^[30,31]^. The 2 intact prophage regions identified in CRC211 (160.5 kb total) are of particular interest. Prophages often carry virulence genes that can be horizontally transferred, potentially enhancing bacterial pathogenicity. For instance, prophage-encoded cytolysins or superantigens could exacerbate tissue damage and chronic inflammation, fostering a tumor-permissive microenvironment. Furthermore, the enrichment of adhesins (e.g., pilus genes) and biofilm-associated proteins suggests that *CRC211* may form microbial communities within tumors, shielding itself from immune clearance and antibiotics. The presence of 75 AMR genes, including those conferring resistance to tetracycline (tetM) and macrolides (ermB), highlights the potential role of *CRC211* in therapy failure among CRC patients. Metabolically, the dominance of amino acid and carbohydrate metabolism pathways is consistent with *CRC211*’s adaptation to utilize tumor-derived nutrient sources, such as lactate and amino acids from necrotic tumor cells. This metabolic flexibility may not only support bacterial survival but also influence tumor cell metabolism through cross-talk, potentially accelerating cancer progression.

Overall, the genomic features of *CRC211* suggest multiple mechanisms through which it may directly influence tumor progression, including persistent colonization that induces chronic inflammation, host DNA damage mediated by genotoxic metabolites, and modulation of local immune responses. Experimental validation is essential to establish causality.

Our findings also point to several potential avenues for clinical translation: (1) Diagnostic Biomarker: The enrichment of *S. oralis* in CRC tissues, along with strain-specific genes or prophage regions in *CRC211*, may aid the development of microbial biomarkers for non-invasive CRC detection using stool or blood-based microbial DNA assays; Phage-Mediated Therapy: (2) The prophage regions identified in *CRC211* could be leveraged for engineered phage therapies that selectively target and lyse CRC-associated *S. oralis*, thereby reducing its pro-tumorigenic effects; (3) Microbiota Modulation: Given its antimicrobial resistance profile and metabolic adaptability, *CRC211* may represent a target for microbiota-modulating interventions, such as probiotics or antibiotics, to restore microbial balance and enhance chemotherapeutic efficacy; (4) Immunotherapy Synergy: Further research could explore whether targeting *CRC211* alters the immune landscape of CRC tumors, potentially synergizing with immune checkpoint inhibitors by reducing immunosuppressive bacterial influences.

Despite providing a genomic foundation for understanding *CRC211*’s role in CRC, several key questions remain: (1) Mechanistic and in vivo validation: The role of *CRC211* in tumorigenesis should be evaluated in *vivo* using gnotobiotic or conventional mouse models of CRC (e.g., AOM/DSS). Colonization with wild-type *CRC211* versus isogenic mutants (e.g., lacking adhesion or biofilm genes) would enable direct assessment of its effects on tumor burden and immune infiltration, moving from association to causality; (2) Host-microbe interactions: Single-cell spatial transcriptomics could reveal *CRC211*'s localization within tumors and its immune-modulatory effects; (3) Therapeutic targeting: Prophage-encoded virulence factors may represent novel targets for microbiota-directed therapies.

In conclusion, the genomic characterization of *S. oralis CRC211* underscores its potential as a CRC-associated bacterium with adaptive traits that promote tumor colonization. These insights not only enhance our understanding of *S. oralis CRC211*’s role in CRC but also provide a foundation for developing microbiota-based diagnostic tools and targeted therapies, contributing to personalized cancer medicine. Future work should integrate in vitro and in vivo models to validate its oncogenic mechanisms and explore its utility as both a diagnostic and therapeutic target.

## DECLARATIONS

### Availability of data and materials

Raw sequencing data have been uploaded to the National Center for Biotechnology Information (NCBI) (Accession GEO number: GSE294724). All data presented in this study are available from the corresponding authors upon reasonable request.

### Copyright

© The Author(s) 2025.
